# Supplementary material for: Parameters of Oxidative and Inflammatory Status in a Three-Month Observation of Patients with Acute Myocardial Infarction Undergoing Coronary Angioplasty—A Preliminary Study
Source: Medicina (Kaunas). 2019 Sep 13;55(9):585. doi: 10.3390/medicina55090585 (PMC6780791; doi:10.3390/medicina55090585)
Supplement: Supplementary file 1 [file medicina-55-00585-s001.pdf]

Supplementary table. Parameters (median with 25-75%) of oxidative and inflammatory status in patients with myocardial infarction (T0) and after 3 months recovery after PTCA (T3) regarding to STEMI and NSTEMI.

Parameter [unit]	T0			T3			STEMI	NSTEMI
	STEMI N=17	NSTEMI N=13	p	STEMI N=17	NSTEMI N=13	p	T0 vs T3 N=17	T0 vs T3 N=13
IMA [ABSU/g]	40.9 (35.8-51.8)	40.0 (42.9-55.5)	0.615	46.4 (41.0-57.3)	47.3 (39.7-52.1)	0.867	0.113	0.311
AOPP [µmol/L]	235.7 (116.1-389.1)	133.9 (104.6-295.8)	0.402	284.9 (179.6-351.2)	261.0 (183.2-390.7)	1.000	0.149	0.196
SH groups [mmol/L]	0.43 (0.37-0.47)	0.42 (0.35-0.43)	0.464	0.40 (0.29-0.50)	0.42 (0.20-0.46)	0.558	0.831	0.196
TAS [µmol/L]	17.2 (15.5-17.8)	17.0 (16.0-18.0)	0.544	16.5 (15.7-17.1)	16.8 (16.2-17.5)	0.690	0.868	0.279
TMAO [µmol/L]	1.20 (0.96-1.27)	1.20 (0.85-1.38)	0.917	1.27 (0.72-1.70)	1.18 (0.81-3.05)	0.305	0.943	0.152
IGF-1 [ng/ml]	122.1 (93.9-163.8)	134.8 (74.6-194.2)	0.933	148.8 (129.6-180.1)	141.2 (85.2-195.0)	0.586	0.753	0.055
Insulin [µU/ml]	16.2 (8.8-29.8)	13.3 (11.2-18.8)	0.967	17.0 (8.3-37.9)	19.0 (14.8-29.8)	0.544	0.943	0.463
hsCRP [mg/L]	3.21 (2.26-7.55)	6.29 (2.51-19.2)	0.217	1.85 (0.51-3.70)	1.16 (0.59-1.94)	0.503	0.084	**0.001**
PSP [mg/L]	2.43 (2.38-2.82)	2.31 (2.08-3.39)	0.543	2.57 (2.23-2.68)	2.48 (2.10-2.75)	0.818	0.943	0.463

Troponin I [μg/L]	15.76<br>(5.85-52.70)	3.74<br>(1.06-11.94)	**0.020**
LV mass [g]	316<br>(278-420)	285<br>(261-411)	0.675
EF [%]	60<br>(55-65)	58<br>(35-60)	0.174

Table legend: IMA – ischemia modified albumin, AOPP - advanced oxidation protein products, SH groups - thiol groups, TAS - total antioxidant status, TMAO - trimethylamine N-oxide, IGF-1 - insulin growth factor-1, hsCRP - high sensitive C-reactive protein, PSP – presepsin. Statistically significant differences are bolded.
